# Risk-adapted locoregional radiotherapy strategies based on a prognostic nomogram for de novo metastatic nasopharyngeal carcinoma patients treated with chemoimmunotherapy

**DOI:** 10.1038/s41598-024-54230-6

**Published:** 2024-02-17

**Authors:** Yuebing Chen, Chuying Chen, Hewei Peng, Shaojun Lin, Jianji Pan, Huiping Zheng, Jingfeng Zong, Cheng Lin

**Affiliations:** 1 Department of Radiation Oncology, Clinical Oncology School of Fujian Medical University, Fujian Cancer Hospital, Fuzhou, Fujian Province China; 2 Department of Epidemiology and Health Statistics, Fujian Provincial Key Laboratory of Environment Factors and Cancer, School of Public Health, Fujian Medical University, Fuzhou, Fujian Province China; 3 Department of Radiation Oncology, Fujian Medical University Xiamen Humanity Hospital, Xiamen, Fujian Province China

**Keywords:** Cancer immunotherapy, Chemotherapy, Radiotherapy

## Abstract

To develop a prognostic nomogram for individualized strategies on locoregional radiation therapy (LRRT) in patients with de novo metastatic nasopharyngeal carcinoma (dmNPC) treated with chemoimmunotherapy. Ninety patients with dmNPC treated with chemoimmunotherapy and diagnosed between 2019 and 2022 were included in our study. Cox regression analysis was performed to identify independent prognostic factors for overall survival (OS) and progression-free survival (PFS) to establish a nomogram. With a median follow-up of 17.5 months, the median PFS and OS were 24.9 months and 29.4 months, respectively. Sixty-nine patients and twenty-one patients were included in the LRRT group and without LRRT group, respectively. Multivariate analysis revealed that younger age, lower EBV DNA copy number before treatment, a single metastatic site, more cycles of chemotherapy and immunotherapy were significantly associated with better OS. A prognostic nomogram was constructed incorporating the above 5 independent factors, with a C-index of 0.894. Patients were divided into low- and high-risk cohorts based on nomogram scores. A significant improvement in OS was revealed in the LRRT group compared with the without-LRRT group for patients in the high-risk cohort (HR = 2.46, 95% CI 1.01–6.00, *P* = 0.049), while the OS was comparable between the two groups in the low-risk cohort. Our study indicates that LRRT may be associated with better prognosis in high-risk patients with dmNPC in the era of immunotherapy.

## Introduction

Nasopharyngeal carcinoma (NPC) has a high prevalence in southeastern Asia^1^. With the development of intensity-modulated radiation therapy (IMRT), the overall survival (OS) rate of patients with early-stage NPC has exceeded 90%^2^. However, de novo metastatic NPCs (dmNPCs) have a poor prognosis, with a nonnegligible proportion of approximately 4–10%^3,4^. Platinum-based chemotherapy is the standard treatment for metastatic NPC, but the 3-year OS rate is only approximately 20–30%^5^. Therefore, identification of optimal treatment strategies that involve combination of radiotherapy and immunotherapy is urgently needed to improve survival outcomes in patients with dmNPC.

Locoregional radiation therapy (LRRT), namely radiotherapy to primary lesions, has been increasingly used to treat metastatic NPC. Several retrospective analyses have illustrated the superiority of LRRT combined with chemotherapy over chemotherapy alone in patients with metastatic NPC^6,7^. More importantly, these results were further validated by a phase 3 randomized clinical trial, which demonstrated significant advantages in terms of OS and progression-free survival (PFS) in dmNPC patients treated with a combination of radiotherapy and chemotherapy^8^. Nevertheless, these studies were conducted without the application of immunotherapy.

The unique immune environment of Epstein–Barr virus (EBV)-associated NPC provides targets for immunotherapy^9^. Compared with chemotherapy alone, chemoimmunotherapy has been shown to improve locoregional control and ultimately OS in three randomized trials recently^10–12^. However, the absolute benefit of PFS was only approximately 3 months, and the objective response rate (ORR) was approximately 70–87%^10–12^, suggesting that chemoimmunotherapy was not sufficient and has yet to be further enhanced. The question arises as to whether LRRT will provide additional survival benefits when combined with chemoimmunotherapy. Since dmNPC is complex and heterogeneous, who should be treated with LRRT is not clear in the era of immunotherapy.

Hence, this study aimed to develop a widely accepted prognostic nomogram for identifying patients who could benefit more from LRRT and thus optimize a risk-adapted therapeutic strategy for dmNPC patients.

## Methods

### Study population

**Figure 1:**
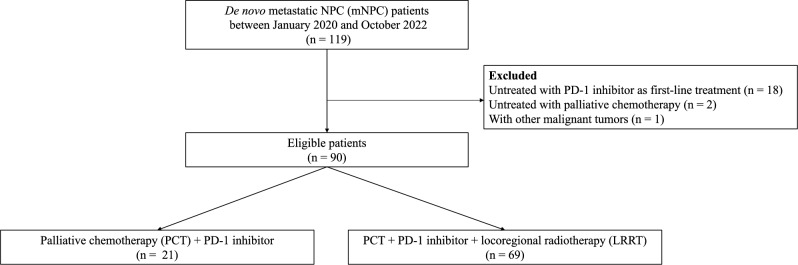
Patient selection flowchart.

Eligible patients with dmNPC between 2019 and 2022 at Fujian Cancer Hospital were retrospectively reviewed (Fig. 1). The inclusion criteria were as follows: (I) pathologically confirmed NPC with initial metastatic disease and (II) received at least one cycle of platinum-based chemotherapy and immunotherapy as first-line systemic therapy. The exclusion criteria for patients were as follows: (I) had other types of cancer and (II) were lost to follow-up.

### Treatment

Patients were staged according to the 8th edition of the American Joint Committee on Cancer Staging System. They received platinum-based chemotherapy and immunotherapy every 3 weeks per cycle until tumor progression, intolerable toxicity, or patient’s refusal of planned chemotherapy for private reasons like economic problems despite recommendations. The main palliative chemotherapy (PCT) regimens included gemcitabine plus platinum (GP), taxane plus platinum (TP), docetaxel plus platinum, 5-fluorouracil (TPF), and platinum plus 5-fluorouracil (PF). Immunotherapy referred to anti-programmed cell death receptor-1 (PD-1) inhibitors, including toripalimab, tislelizumab, sintilimab, camrelizumab, and pembrolizumab. The choice of LRRT or local treatment for metastatic lesions was at the discretion of the radiation oncologist. LRRT to the nasopharynx and neck was conducted as described previously^13^. In brief, clinical target volume1 (CTV1) was defined as the high-risk region, and CTV2 included potentially involved regions and the retropharyngeal nodal regions. Radiation dose included a total dose of 66–70.95 Gy to the planning target volume (PTV) of GTV (gross target volume), 60-66 Gy to the PTV of CTV-1, and 54–55 Gy to PTV of CTV-2 and CTV-N. A total of 28.9% (26/90) of patients received local therapy for metastatic lesions, such as bone, liver, and lung metastatic lesions. Among them, 16 patients with bone metastasis received radiotherapy with a dose of 20–50 Gy. For patients with liver metastasis, 77.8% (7/9) were treated with radiofrequency ablation, and the remaining of them received transarterial chemoembolization and radiotherapy. And one NPC patient with lung metastasis underwent lobectomy.

PFS was defined as the time interval from diagnosis to the first defined events, including locoregional recurrence, distant metastasis relapse, or death from any cause. OS was calculated from the date of diagnosis to the date of recorded death or the last follow-up.

### Statistical analysis

All the statistical analyses were performed using SPSS statistical software V.24.0 (Chicago, USA), GraphPad Prism 8 (GraphPad Prism, USA), and *R* software (version 4.2.1). Clinicopathological characteristics were compared between the LRRT group and the without-LRRT group using the χ^2^ test. Receiver operating characteristic (ROC) curve was used for the optimal cutoff value of EB-DNA levels and the cycle of first-line immunotherapy (Supplementary Fig. S1). Survival curves were estimated and compared by the Kaplan‒Meier method and log-rank test. Multivariate analyses were performed using the Cox proportional hazards model to identify independent prognostic factors. The time-dependent area under the curve (AUC) of the ROC curves, C-index, and calibration map were calculated to evaluate the predictive and discriminative ability of the nomogram. A two-sided *P* < 0.05 was considered to indicate statistical significance.

### Ethical approval and consent to participate

This study was approved by the Ethical Review Committee of Fujian Cancer Hospital (Approval No. K2023-046-01) and carried out in accordance with relevant guidelines and regulations. All patients included in this study signed informed consent to the treatment protocol statements.

## Results

### Patient characteristics and treatment outcomes

A total of 90 patients were included in the study (Table 1). All patients were undifferentiated nonkeratinizing NPC. With a median follow-up of 17.5 (4.6 to 52.7) months, the median PFS was 24.9 months, and the median OS was 29.4 months. The 1-year and 2-year OS and PFS were 93% and 65.4% and 67.9% and 52.5%, respectively.

**Table 1:** Baseline characteristics of the study population. ECOG Eastern Cooperative Oncology Group.

Characteristic	LRRT, n (%)	Without-LRRT, n (%)	*P*
Age (year)			1.000
< 50	35 (50.7)	11 (52.4)	
≥ 50	34 (49.3)	10 (47.6)	
Gender			1.000
Male	56 (81.2)	17 (81.0)	
Female	13 (18.8)	4 (19.0)	
ECOG score			0.233
0	0 (0)	1 (4.8)	
1	69 (100)	20 (95.2)	
T stage			0.771
T1-2	17 (24.6)	4 (19.0)	
T3-4	52 (75.4)	17 (81.0)	
N stage			0.506
N0-1	12 (17.4)	2 (9.5)	
N2-3	57 (82.6)	19 (90.5)	
Pre-treatment EBV DNA			0.121
< 66,600	47 (68.1)	10 (47.6)	
≥ 66,600	22 (31.9)	11 (52.4)	
Multiple organ metastasis			0.012
No	42 (60.9)	6 (28.6)	
Yes	27 (39.1)	15 (71.4)	
No. of metastatic lesions			0.177
Single	14 (20.3)	1 (4.8)	
Multiple	55 (79.7)	20 (95.2)	
Liver metastases			0.006
No	47 (68.1)	7 (33.3)	
Yes	22 (31.9)	14 (66.7)	
Bone metastases			0.430
No	24 (34.8)	5 (23.8)	
Yes	45 (65.2)	16 (76.2)	
Lung metastases			0.792
No	47 (68.1)	13 (61.9)	
Yes	22 (31.9)	8 (38.1)	
Lactate dehydrogenase (U/L)			0.036
< 250	44 (67.7)	8 (40.0)	
≥ 250	21 (32.3)	12 (60.0)	
Cycle of first-line chemotherapy			0.202
< 6	24 (34.8)	11 (52.4)	
≥ 6	45 (65.2)	10 (47.6)	
Cycle of first-line immune therapy			0.040
< 8	37 (53.6)	17 (81.0)	
≥ 8	32 (46.4)	4 (19.0)	
Local treatment to metastasis			0.005
No	44 (63.8)	20 (95.2)	
Yes	25 (36.2)	1 (4.8)	

**Figure 2:**
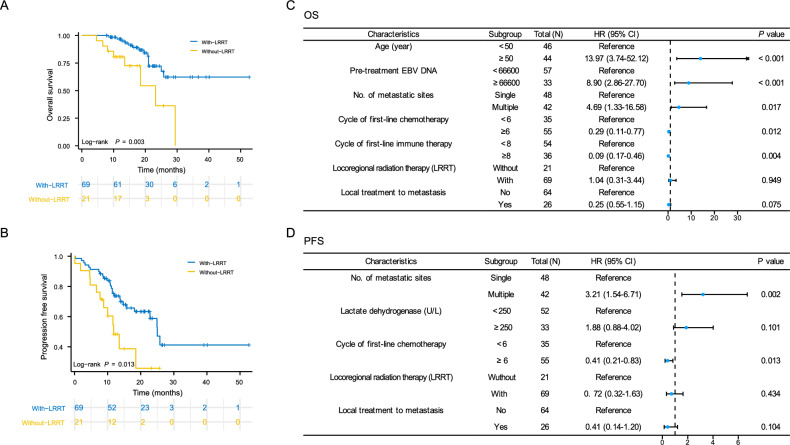
Kaplan‒Meier curves of overall survival (**A**) and progression-free survival (**B**) in LRRT and without-LRRT group; Forest plot of multivariate Cox regression analysis for overall survival (**C**) and progression-free survival (**D**).

Of these, 69 (76.7%) and 21 (23.3%) patients received PCT plus immunotherapy with or without LRRT, respectively. Compared to those in the LRRT group, patients in the without-LRRT group were more likely to have multiple organ metastasis and liver metastasis. In addition, the proportion of patients who received more than 8 cycles of immunotherapy and local treatment for metastatic lesions was greater in the LRRT group than in the without-LRRT group. Kaplan‒Meier analysis showed that patients in the LRRT group had significantly better 2-year OS (72.3 vs. 36.3%, *P* = 0.003) and PFS (58.9 vs. 25.8%, *P* = 0.013) than those in the without-LRRT group (Fig. 2A, B).

### Construction and validation of the prognostic nomogram

**Figure 3:**
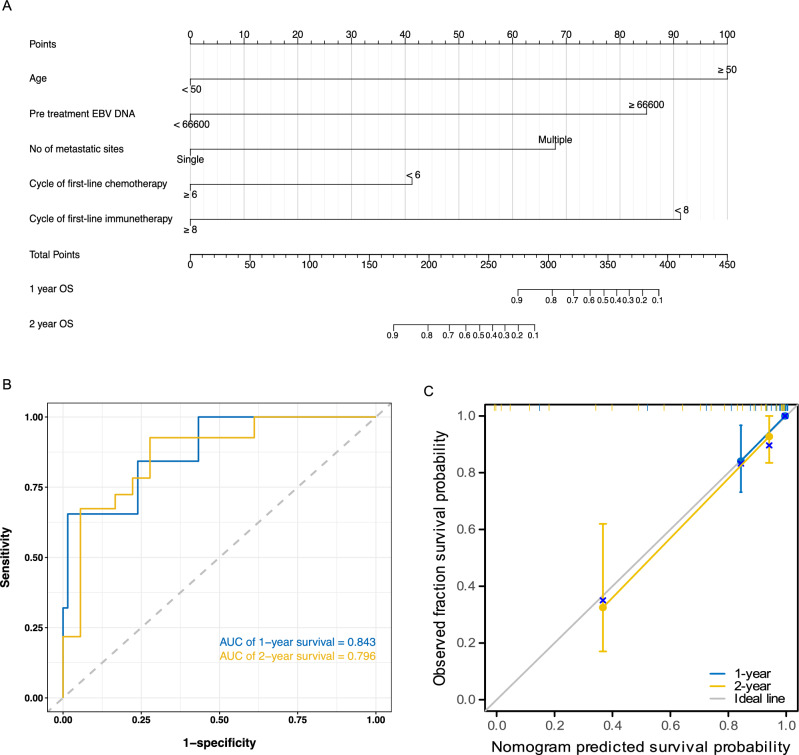
Nomogram for predicting 1- and 2-year overall survival in patients with de novo metastatic nasopharyngeal carcinoma treated with chemoimmunotherapy (**A**), receiver operating characteristic curve (**B**) and calibration curve (**C**).

The results of univariate analysis are shown in Supplementary Table S1. According to our multivariate analysis, younger age (≤ 50 years), a lower EBV DNA copy number (< 66,600 copies before treatment), a single metastatic site, more cycles of chemotherapy (≥ 6 cycles), and immunotherapy (≥ 8 cycles) were identified as independent prognostic factors associated with better OS (Fig. 2C, Supplementary Fig. S1). In addition, a single metastatic site and more cycles of chemotherapy (≥ 6 cycles) were favorable for PFS according to multivariate analysis (Fig. 2D, Supplementary Fig. S2). Since, OS is the gold standard of survival benefits, the 5 prognostic factors above were subsequently included in our established nomogram for OS (Fig. 3A). One example of how to use the nomogram was shown in Supplementary Fig. S3. The prognostic nomogram showed good accuracy in predicting OS, with a C-index of 0.894 (95% CI 0.829–0.958), a 1-year AUC of 0.843 and a 2-year AUC of 0.796 (Fig. 3B). In addition, the calibration plots showed excellent consistency between the actual and nomogram-predicted survival probabilities (Fig. 3C).

### Risk stratification

**Figure 4:**
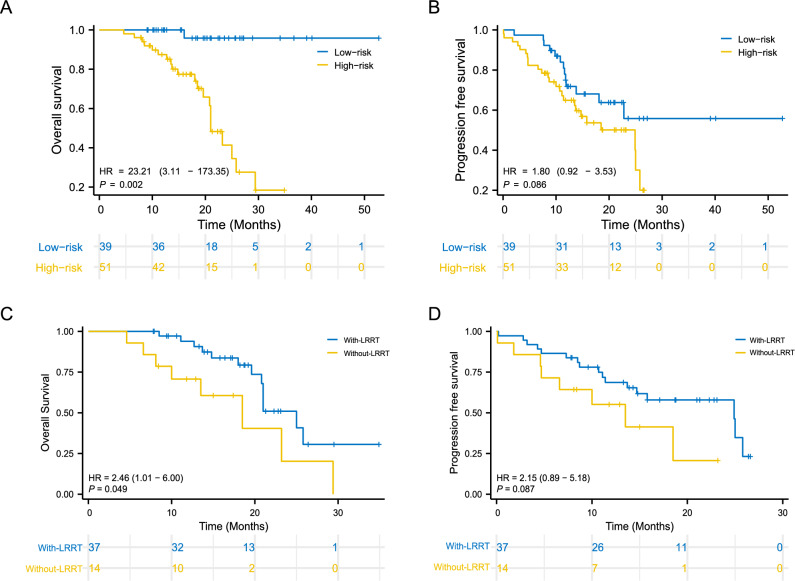
Comparisons of overall survival and progression-free survival curves between the low-risk cohort and high-risk cohort (**A**, **B**); Comparisons of overall survival and progression-free survival curves between the LRRT and without-LRRT groups in the high-risk cohort (**C**, **D**).

Based on the mean value of nomogram scores, all patients were divided into two risk cohorts: the low-risk group with score below average (n = 39) and the high-risk group with score above average (n = 51). The baseline characteristics of the high-risk and low-risk patients are shown in Table 2. Clinicopathological characteristics were well balanced between the LRRT and non-LRRT groups in the low-risk cohort, while more patients with liver metastases and who received local treatment for metastases were in the non-LRRT group than in the LRRT group in the high-risk cohort. Patients in the low-risk cohort had significantly better 2-year OS than did those in the high-risk cohort (95.8 vs. 41.4%, *P* = 0.002); however, no statistically significant difference was found in PFS between the 2 cohorts (Fig. 4A–B). Subgroup analysis of patients in the LRRT cohort revealed significant improvements in OS compared with those in the without-LRRT subgroup for patients in the high-risk cohort (*P* = 0.049) (Fig. 4C–D); however, there were no significant differences in OS or PFS between the LRRT and without-LRRT groups in the low-risk cohort.

**Table 2:** The baseline variables of the patients in each risk cohort.

Variables	Low-risk cohort			High-risk cohort		
	LRRT, n (%)	Without-LRRT, n (%)	*P*	LRRT, n (%)	Without-LRRT, n (%)	*P*
Age (year)			0.588			0.166
< 50	27 (84.4)	5 (71.4)		8 (57.1)	6 (42.9)	
≥ 50	5 (15.6)	2 (28.6)		29 (78.4)	8 (42.9)	
Gender			1.000			0.692
Male	25 (78.1)	6 (85.7)		31 (83.8)	11 (78.6)	
Female	7 (21.9)	1 (14.3)		6 (16.2)	3 (21.4)	
T stage			1.000			0.739
T1-2	7 (21.9)	1 (14.3)		10 (27.0)	3 (21.4)	
T3-4	25 (78.1)	6 (85.7)		27 (73.0)	11 (78.6)	
N stage			1.000			0.657
N0-1	6 (18.8)	1 (14.3)		6 (16.2)	1 (7.1)	
N2-3	26 (81.3)	6 (85.7)		31 (83.8)	13 (92.9)	
Pre-treatment EBV DNA			1.000			0.116
< 66,600	26 (81.3)	6 (85.7)		21 (56.8)	4 (28.6)	
≥ 66,600	6 (18.8)	1 (14.3)		16 (43.2)	10 (71.4)	
Multiple organ metastasis			0.205			0.064
No	20 (62.5)	2 (28.6)		22 (59.5)	4 (28.6)	
Yes	12 (37.5)	5 (71.4)		15 (40.5)	10 (71.4)	
No. of metastatic lesions			0.653			0.305
Single	9 (28.1)	1 (14.3)		5 (13.5)	0 (0)	
Multiple	23 (71.9)	6 (85.7)		31 (86.5)	14 (100)	
Liver metastases			0.379			0.013
No	24 (75.0)	4 (57.1)		23 (62.2)	3 (21.4)	
Yes	8 (25.0)	3 (42.9)		14 (37.8)	11 (78.6)	
Bone metastases			0.666			0.522
No	8 (25.0)	1 (14.3)		16 (43.2)	4 (28.6)	
Yes	24 (75.0)	6 (85.7)		21 (56.8)	10 (71.4)	
Lung metastases			0.654			1.000
No	23 (71.9)	4 (57.1)		24 (64.9)	9 (64.3)	
Yes	9 (28.1)	3 (42.9)		13 (35.1)	5 (35.7)	
Lactate dehydrogenase (U/L)			0.596			0.114
< 250	24 (80.0)	4 (66.7)		20 (57.1)	4 (28.6)	
≥ 250	6 (20.0)	2 (33.3)		15 (42.9)	10 (71.4)	
Cycle of first-line chemotherapy			1.000			0.225
< 6	5 (15.6)	1 (14.3)		19 (51.4)	10 (71.4)	
≥ 6	27 (84.4)	6 (85.7)		18 (49.6)	4 (28.6)	
Cycle of first-line immunotherapy			0.344			0.169
< 8	7 (21.9)	3 (42.9)		30 (81.1)	14 (100)	
≥ 8	25 (78.1)	4 (57.1)		7 (18.9)	0 (0)	
Local treatment to metastasis			0.388			0.011
No	20 (62.5)	6 (85.7)		24 (64.9)	14 (100)	
Yes	12 (37.5)	1 (14.3)		13 (35.1)	0 (0)

## Discussion

The use of LRRT for treating dmNPC is an important topic that has not been resolved due to the lack of randomized trials in the era of immunotherapy. To the best of our knowledge, our study is the first to create a risk stratification nomogram to explore the value of LRRT in dmNPC patients treated with chemoimmunotherapy. Our results showed that LRRT may be associated with improved OS in the high-risk cohort, while no survival benefit was found between the LRRT group and the without-LRRT group in the low-risk cohort.

The effect of LRRT on metastatic NPC after treatment with platinum-based chemotherapy was significant^6–8^. Recently, immunotherapy combined with chemotherapy was recommended as a first-line treatment for dmNPC^14^ based on multiple phase III randomized clinical trials^10–12^. However, whether the addition of LRRT is still indispensable in the era of immunotherapy needs further exploration. Hu and Liu et al. revealed that LRRT following chemoimmunotherapy improved survival outcomes in selective dmNPC patients, as indicated by undetectable EBV DNA levels after immunotherapy, oligometastases, and good response after chemoimmunotherapy^15,16^. Our results yielded similar conclusions, as we observed better 2-year OS and PFS in the LRRT group. However, dmNPC is highly heterogeneous^17^, and a single factor is limited in its ability to predict efficacy and prognosis because all clinicopathological prognostic factors need to be considered, as does the proportional influence of these factors. Consequently, the nomogram we developed that incorporated independent prognostic factors was more effective at facilitating individualized decision-making. Our analysis proposed that high-risk dmNPC patients, rather than low-risk patients, may benefit from LRRT, thus avoiding undertreatment and overtreatment.

However, the mechanism underlying the effect of radiation at the primary site on the overall disease trajectory in patients with metastatic NPC is still unclear. One viewpoint is that irradiation, especially hypofractionated radiotherapy, may play an important role in stimulating systemic antitumor responses and thus have an abscopal effect, and this effect is enhanced when immunotherapy is added to the immune system^18–20^. However, whether the abscopal effect could explain the benefit of LRRT is unknown, as patients in the LRRT group received low-dose fractionated radiotherapy in our study^21^. Furthermore, patients with high-risk factors, including higher levels of plasma EBV-DNA before treatment and multiple metastatic sites, could benefit from the combined therapy in our study, suggesting the potential benefit of treatment for patients with a high tumor burden^22^. The reason may be that chemoimmunotherapy alone is an undertreatment for patients with a high tumor burden. Notably, more patients in the LRRT group than in the without LRRT group received local treatment for metastases in the high-risk cohort. However, whether local treatment for metastatic lesions would improve survival is still controversial^23,24^. The results underscore the importance of LRRT in the high-risk cohort.

mNPC patients with liver metastases were supposed to be associated with worse outcomes, however, whether aggressive treatment was considered in those patients remains controversial^7,25–27^. Zou et al. reported that systemic chemotherapy combined with LRRT was not beneficial in de novo mNPC with liver metastases regardless of metastatic lesions. While, pan et al. revealed that mNPC patients with limited liver metastases or unilobular metastases may live longer and more aggressive therapy was recommended^25^. At present, for mNPC patients, LRRT after palliative chemotherapy was suggested in oligometastatic patients and chemotherapy-sensitive patients^8,28^. As to the treatment of liver metastases, radiofrequency ablation was supposed to improve survival in selected patients^29^. Therefore, further validation of the prognostic model is needed. It was worth mentioning that there were more patients with liver metastases in the without-LRRT subgroup than in the LRRT subgroup in the high-risk cohort, which could lead to bias. The survivor treatment selection bias was inevitable and difficult to overcome as patients who were selected for LRRT tended to be with favorable risk factors and survive long enough in clinical practice. The decision to treat aggressively or not was affected by many limitations, such as patients' tolerance for therapy, the presence of comorbidity, disease progression and socio-economic factors. Thus, our observed survival benefit between LRRT and without LRRT group might reflect the true consequence of mNPC. Future studies to confirm the results will take this into consideration.

The timing, sequencing, and treatment cycles between radiation and chemoimmunotherapy are common practical questions considering toxicity and therapeutic outcomes. There was a lack of recording information on toxicity in our study, but well-tolerated LRRT concurrent with immunotherapy has been shown in other studies^15,30^. We found that receiving more than 8 cycles of immunotherapy was a favorable prognostic factor in dmNPC patients. Currently, the duration of immunotherapy differs in the setting of previous clinical trials^31,32^. Studies have suggested that chemoimmunotherapy followed by LRRT with concurrent immunotherapy may lead to longer PFS^16,33^. Hence, chemoimmunotherapy combined with sequential LRRT combined with immunotherapy is at least safe and favorable for ensuring a good prognosis, while the duration of immunotherapy still needs further research.

There are several limitations in our study. Selection bias was inevitable due to the retrospective design. In addition, a major proportion of histology in endemic areas is undifferentiated nonkeratinizing NPC^34^, which limits the use of our prognostic model in keratinizing NPC in nonendemic areas. Besides, the effect of local treatment of metastatic lesions was not evaluated, as only a small proportion of patients received this treatment in our study. And due to limited sample size in the low-risk group, further analysis was not conducted, which is our future direction to explore how to improve survival in low-risk patients by other therapies. A larger population and longer follow-up period are needed to confirm our results.

## Conclusion

Our study indicates that LRRT may be associated with better prognosis in dmNPC patients receiving chemoimmunotherapy, especially in those with high-risk factors. However, further studies are needed to verify and improve the prognostic model.

### Supplementary Information

Supplementary Information.

## Data Availability

The datasets generated for this study are available on request to the corresponding author.
