# Supplementary material for: Risk-adapted locoregional radiotherapy strategies based on a prognostic nomogram for de novo metastatic nasopharyngeal carcinoma patients treated with chemoimmunotherapy
Source: Sci Rep. 2024 Feb 17;14:3950. doi: 10.1038/s41598-024-54230-6 (PMC10873310; doi:10.1038/s41598-024-54230-6)
Supplement: Supplementary file 1 — Supplementary Information. [file 41598_2024_54230_MOESM1_ESM.docx]

**Supplemental materials**

**
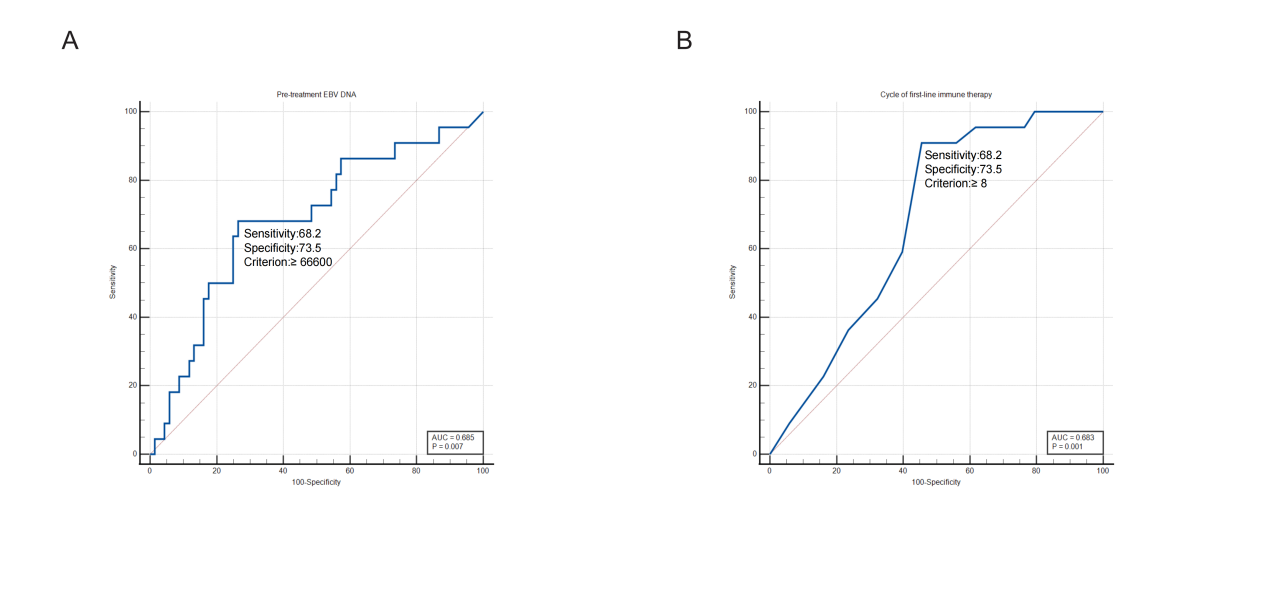
**

**Supplementary** **Figure S1** ROC curves for pre-treatment EBV DNA copies (A) and cycle of first-line immunotherapy (B)


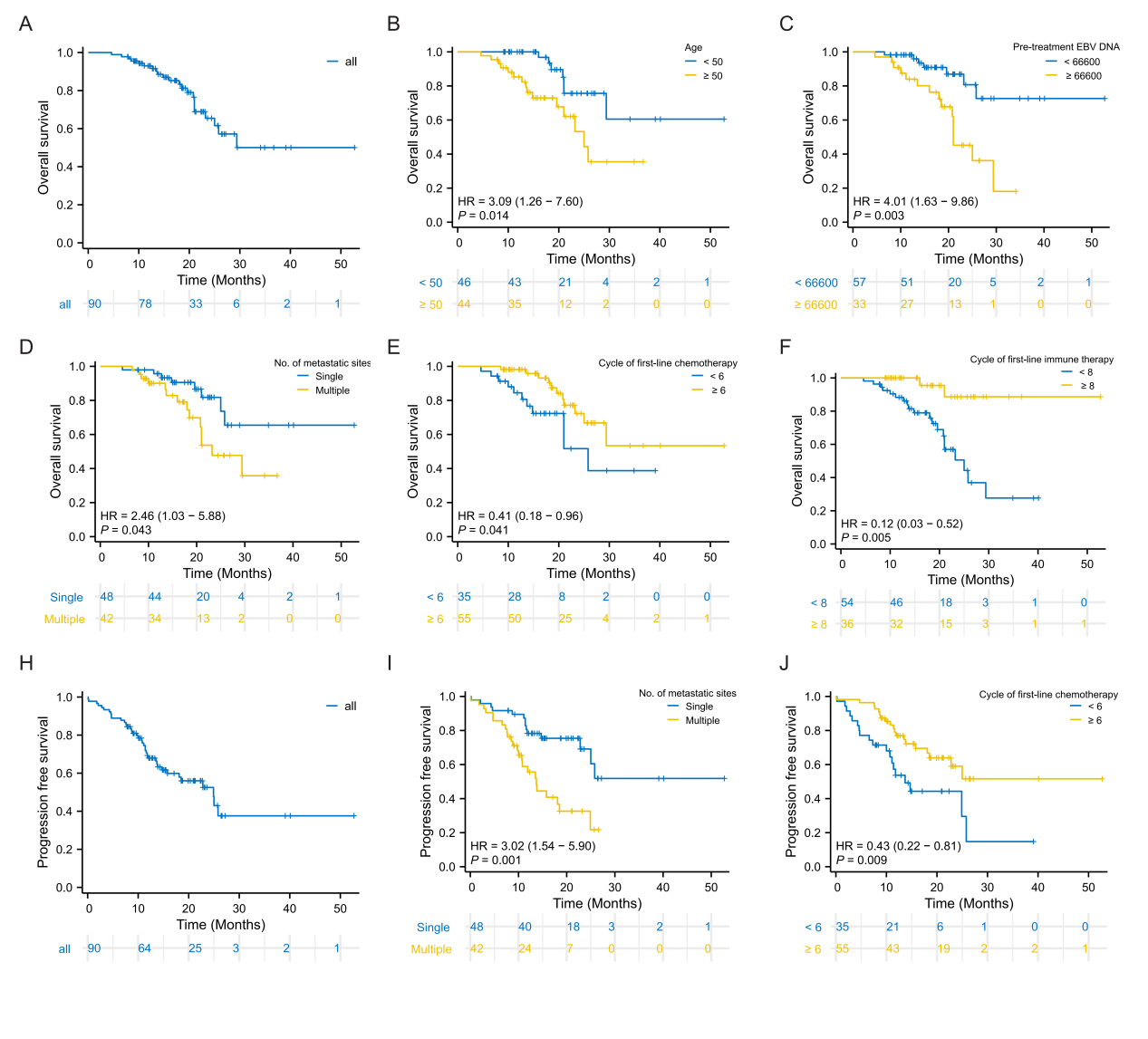


**Supplementary** **Figure S2** Overall survival (OS) in the whole cohort (A); Comparisons of OS between groups: age < 50 versus ≥ 50 years (B); EBV DNA < 66600 copies versus ≥ 66600 copy nnumbers before treatment (C); single metastatic site versus multiple metastatic sites (D); < 6 versus ≥ 6 cycles of chemotherapy (E); < 8 versus ≥ 8 immunotherapy regimens (F); Progression free survival (PFS) in the whole cohort (H); Comparisons of PFS between groups: single metastatic site versus multiple metastatic sites (I); < 6 versus ≥ 6 cycles of chemotherapy (J).


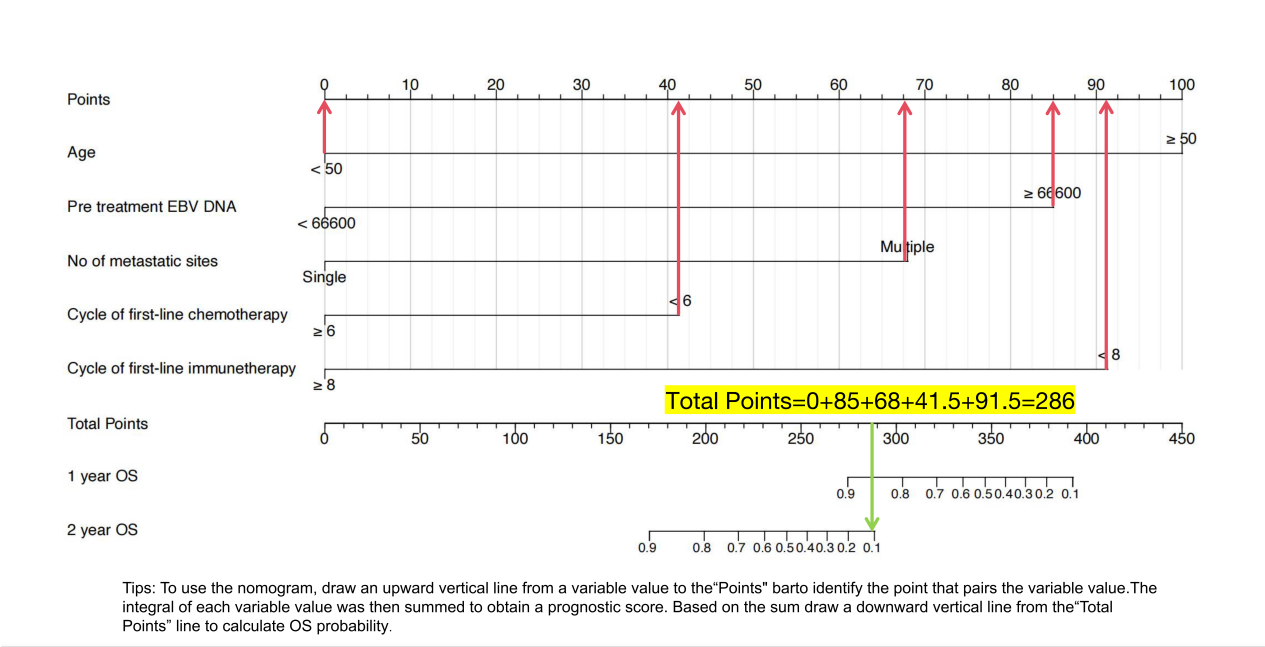


**Supplementary** **Figure S3** The example of how to use the nomogram

| **Variables** | **OS** | | **PFS** | |
| --- | --- | --- | --- | --- |
|  | **HR (95%CI）** | ***P*** | **HR (95%CI）** | ***P*** |
| Age (year) |  |  |  |  |
| < 50 | Reference |  | Reference |  |
| ≥ 50 | 3.10 (1.26-7.62) | 0.014 | 1.13 (0.60-2.15) | 0.707 |
| Gender |  |  |  |  |
| Male | Reference |  | Reference |  |
| Female | 1.42 (0.42-4.84) | 0.575 | 0.94 (0.43-2.05) | 0.874 |
| T stage |  |  |  |  |
| T1-2 | Reference |  | Reference |  |
| T3-4 | 1.04 (0.35-3.08) | 0.944 | 0.84 (0.40-1.78) | 0.647 |
| N stage |  |  |  |  |
| N0-1 | Reference |  | Reference |  |
| N2-3 | 2.19 (0.51-9.38) | 0.292 | 1.11 (0.46-2.66) | 0.814 |
| Pre-treatment EBV DNA |  |  |  |  |
| < 66600 | Reference |  | Reference |  |
| ≥ 66600 | 3.95 (1.61-9.72) | 0.003 | 1.79 (0.94-3.38) | 0.075 |
| Multiple organ metastasis |  |  |  |  |
| No | Reference |  | Reference |  |
| Yes | 2.45 (1.02-5.84) | 0.044 | 3.02 (1.54-5.91) | 0.001 |
| No. of metastatic lesions |  |  |  |  |
| Single | Reference |  | Reference |  |
| Multiple | 4.41 (0.59-32.81) | 0.148 | 3.14 (0.96-10.25) | 0.058 |
| Liver metastases |  |  |  |  |
| No | Reference |  | Reference |  |
| Yes | 2.01 (0.86-4.70) | 0.106 | 1.79 (0.94-3.38) | 0.076 |
| Bone metastases |  |  |  |  |
| No | Reference |  | Reference |  |
| Yes | 1.34 (0.52-3.42) | 0.542 | 1.44 (0.70-2.96) | 0.327 |
| Lung metastases |  |  |  |  |
| No | Reference |  | Reference |  |
| Yes | 1.76 (0.76-4.09) | 0.191 | 1.58 (0.82-3.04) | 0.172 |
| Lactate dehydrogenase (U/L) |  |  |  |  |
| <250 | Reference |  | Reference |  |
| ≥250 | 1.58 (0.65-3.83) | 0.315 | 1.95 (1.00-3.80) | 0.049 |
| Cycle of first-line chemotherapy |  |  |  |  |
| <6 | Reference |  | Reference |  |
| ≥6 | 0.42 (0.18-0.97) | 0.043 | 0.43 (0.22-0.81) | 0.009 |
| Cycle of first-line immunotherapy |  |  |  |  |
| <8 | Reference |  | Reference |  |
| ≥8 | 0.12 (0.03-0.53) | 0.005 | 0.80 (0.42-1.56) | 0.516 |
| Local treatment to metastasis |  |  |  |  |
| No | Reference |  | Reference |  |
| Yes | 0.21 (0.05-0.92) | 0.038 | 0.31 (0.12-0.80) | 0.015 |

**Supplementary** **Table S1** Univariate analysis of 90 patients with *de novo* metastatic NPC.
